# First Report on Successful Conversion of Long-Term Treatment of Recurrent Atypical Hemolytic Uremic Syndrome With Eculizumab to Ravulizumab in a Renal Transplant Patient

**DOI:** 10.3389/ti.2022.10846

**Published:** 2022-10-03

**Authors:** Ulrich Jehn, Ugur Altuner, Hermann Pavenstädt, Stefan Reuter

**Affiliations:** Department of Medicine D, Division of General Internal Medicine, Nephrology and Rheumatology, University Hospital of Münster, Münster, Germany

**Keywords:** kideny transplantation, ravulizumab, eculizumab, atypical hemolytic uremic syndrome, C5 inhibition

Dear Editors,

Ravulizumab is a long-acting C5-complement monoclonal antibody developed through targeted modifications of eculizumab to significantly extend the half-life of the drug with comparable affinity and specificity to eculizumab (approx. 52 days vs. approx. 11 days) [1]. The efficacy and safety of ravulizumab in patients with aHUS treated with or without complement inhibitors has been adequately studied in adults [2] and pediatric patients [3] and recently led to the approval of the drug by the European Medicines Agency and the US Food and Drug Administration (Ultomiris^®^ SmPC). During 26 weeks of treatment, ravulizumab provided rapid and effective complement inhibition with no unexpected safety issues.

In renal transplant patients, there has been only a single report of ravulizumab use. Ravulizumab was successfully administered in the case of a living kidney donation in a patient with aHUS over the reported treatment period of 6 months [4].

Here we report the results of a young woman who was successfully switched from chronic aHUS treatment with eculizumab to ravulizumab after kidney transplantation.

Back in 2013, we published on the long-term eculizumab treatment of a kidney transplant patient who had a relapse of her aHUS shortly after a living kidney donation [5]. The cause of the aHUS relapse was an MCP mutation and, as was determined in a later analysis, also a factor H mutation. Recurrent aHUS attributable to both complement factor mutations requires lifelong anti-C5 treatment due to high risk [6]. Our patient had been treated with eculizumab administered every 14 days for more than 10 years. As shown in Figure 1, the complete available laboratory data of creatinine, hemoglobin, and platelets show a very stable course of the patient. Remarkably, only one episode of fever occurred during the entire observation period, the cause of which remained unclear. However, the patient achieved *restitutio ad integrum* with short-term inpatient treatment with piperacillin/tazobactam. Because eculizumab has been shown to be effective after renal transplantation for treatment of aHUS and because ravulizumab is a modified version of eculizumab, we expected comparable efficacy and safety of both products [7]. Immunosuppressive therapy consisted of tacrolimus (target through 4–6 ng/ml), low dose mycophenolate mofetil, and prednisolone. At the time of conversion, our 39-year-old patient (body weight 70 kg, BMI 19.6 kg/m^2^) had a serum creatinine of 1.66 mg/dl (eGFR 39 ml/min/1.73 m^2^ (CKD-EPI formula)), hemoglobin concentration of 11.6 g/dl, and platelet count of 359 × 10^3^/mm^3^). After 22 months of therapy with ravulizumab 3,300 mg every 8 weeks following an induction therapy with additional administration of 3,300 mg 2 weeks after the first infusion according to the prescribing information, serum creatinine [1.63 mg/dl (eGFR 39 ml/min/1.73 m^2^)], hemoglobine (12.6 g/dl), and platelet count (367 × 10^3^/mm^3^) were stable over time (Figure 1). However, 14 months after conversion, SARS-CoV-2 infection was diagnosed out-of-hospital between two infusion appointments without our knowledge. The patient, who had been vaccinated three times had severe illness lasting 10 days, but without respiratory distress or graft failure. The patient’s migraine was not changed by the switch to ravulizumab.

**FIGURE 1:**
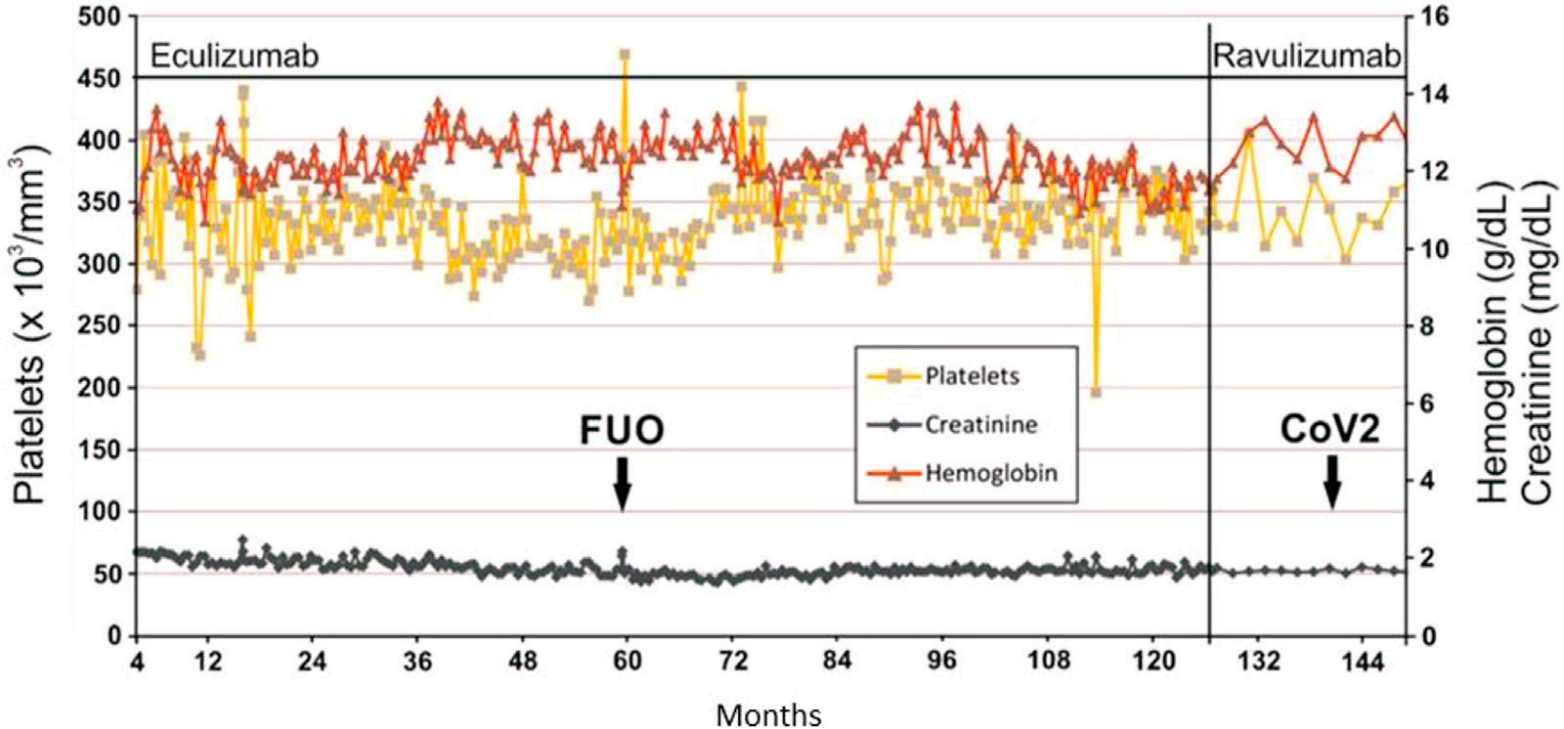
Laboratory values under complement inhibition with eculizumab or ravulizumab. FUO, fever of unknown origin; CoV2, SARS CoV2 infection.

We present this case report because ravulizumab therapy offers improvement in health-related quality of life and greater cost-effectiveness compared with eculizumab therapy because of the longer interval between infusions [8]. The presented case demonstrates that switching C5 inhibition to ravulizumab is safe and effective in renal transplant patients with genetic aHUS, even after decades of therapy with eculizumab. It should be noted that meningococcal vaccination or prophylaxis must be continue with ravulizumab administration (Ultomiris^∗^ SmPC). Because ravulizumab-based therapy offers significant health-related quality of life and cost-effectiveness benefits, it may be the therapy of choice for these patients.

## Data Availability

The original contributions presented in the study are included in the article/supplementary material, further inquiries can be directed to the corresponding author.
